# Evaluation of the antidiabetic potential and bioaccessibility of propolis-enriched aronia kombucha: an *in vitro* study

**DOI:** 10.3389/fnut.2026.1819568

**Published:** 2026-04-22

**Authors:** Gözde Alkan, Seydi Yıkmış, Melikenur Türkol, Emad Karrar, Moneera O. Aljobair, Isam A. Mohamed Ahmed, Suleiman A. Althawab

**Affiliations:** 1 Nutrition and Dietetics, Faculty of Health Sciences, Tekirdag Namik Kemal University, Tekirdag, Türkiye; 2 Department of Food Technology, Tekirdag Namik Kemal University, Tekirdag, Türkiye; 3 Department of Plant Sciences, North Dakota State University, Fargo, ND, United States; 4 Department of Sports Health, College of Sports Sciences and Physical Activity, Princess Nourah bint Abdulrahman University, Riyadh, Saudi Arabia; 5 Department of Food Sciences and Nutrition, College of Food and Agricultural Sciences, King Saud University, Riyadh, Saudi Arabia

**Keywords:** antidiabetic activity, aronia, kombucha, phenolic compounds, propolis

## Abstract

Defined as a fermented tea beverage, kombucha is obtained via the metabolic activity of a symbiotic culture of bacteria and yeasts (SCOBY) in sweetened tea, and it is widely recognized for its associated functional and bioactive properties. In this study, kombucha fermented with aronia tea (AK) and aronia kombucha enriched with propolis (PAK) were examined together to evaluate bioactive components, phenolic profile, *in vitro* bioaccessibility, and antidiabetic potential. Response surface methodology showed that the amount of aronia tea (X_1_) and the concentration of propolis (X_2_) had significant effects on TPC, TFC, and DPPH; the optimum formulation was obtained at a level of 11.09 g/L aronia tea and 1.42% propolis. Throughout the fermentation, PAK exhibited higher values for TPC compared to AK; TPC, which was 367.32 ± 2.56 μg GAE/mL on day 0, was maintained at 345.28 ± 4.40 μg GAE/mL on day 14. Although TPC/TFC decreased in both groups during simulated digestion, PAK maintained a higher level in all phases; recovery was found to be ~34% in TPC and ~40% in TFC. In the phenolic profile, rutin (1.18 → 3.03 μg/mL) and t-ferulic acid (0.66 → 2.21 μg/mL) increased with fermentation, while the addition of propolis significantly enriched flavonoids, especially chrysin (≈17.75–19.14 μg/mL) and quercetin (day 14: 1.55 μg/mL). This phenolic potentiation is consistent with PAK enhancing *α*-glucosidase (42.04%) and α-amylase (44.68%) inhibition. Future studies should investigate reproducibility, colonic fermentation-microbiota interaction, and target metabolite tracking under varying sucrose levels and SCOBY profiles; clinical validation should also be supported by sensory acceptance, shelf life, and safety parameters.

## 1 Introduction

Kombucha is generated by fermenting sucrose-enriched black tea with a symbiotic bacterial–yeast community (SCOBY), which drives the biotransformation process (1). Kombucha has two distinct phases within the beverage: the liquid portion and the biofilm layer on the surface (2). While its origins trace back to China, it has gained increasing global attention in recent years due to its potential health benefits (3). Over time, different alternative substrates such as honey, molasses, and coffee have begun to be used in kombucha production with the aim of obtaining a more health-enriched formulation. Additionally, fermentation aids such as plant extracts, bee pollen are also preferred in the development of new formulations to enrich the biological activity and sensory properties of kombucha (4). Kombucha has numerous health effects such as antioxidant, antimicrobial, anti-inflammatory, antidiabetic, antihypertensive, anticancer, hepatoprotective and detoxifying effects (5). Fermentation in kombucha involves the production of enzymes, organic acids, and various secondary metabolites by microorganisms, which collectively contribute to the beverage’s functional characteristics. This drink contains a symbiotic consortium of yeasts and acetic acid bacteria. Yeasts primarily metabolize the added sucroses in tea, converting them into ethanol and intermediate metabolites, while acetic acid bacteria utilize ethanol during fermentation and contribute to the formation of cellulose-based structures (6). During kombucha fermentation, microorganisms form a cellulose-rich biofilm that has recently gained considerable attention in the food industry due to its unique structural properties and versatile application potential (7).

*Aronia melanocarpa*, commonly known as black chokeberry, is a plant native to the eastern regions of North America. It belongs to the Maloideae subfamily of the Rosaceae family. Although the sour taste of the fruit limits its fresh consumption, it is frequently used in the food industry for the production of fruit juice, tea, wine, jam, jelly, and supplement products (8). Black chokeberry (*Aronia melanocarpa*) is a rich source of polyphenols, containing flavonoids such as anthocyanins, flavanols, and flavonols, as well as various phenolic acids, primarily chlorogenic acid. These compounds exhibit protective and health-promoting properties against many diseases in human health (9). It has been reported to demonstrate a broad spectrum of bioactive properties that may confer health benefits, including antidiabetic, anti-infective, anti-obesity, and antioxidant activities, along with cardioprotective, hepatoprotective, and neuroprotective effects (10).

Propolis is a complex natural substance primarily consisting of lipophilic compounds, beeswax, essential oils, pollen grains, and a wide range of organic constituents. Depending on its botanical origin, it is commonly classified into green, red, and brown varieties, each characterized by distinct phytochemical compositions. These types differ notably in their content of flavonoids, phenolic acids, terpenoids, chalcones, sesquiterpenes, and other biologically active metabolites. Extensive research has demonstrated that propolis possesses diverse pharmacological activities, including antidiabetic, anti-inflammatory, antioxidant, and anticancer effects. Moreover, it has been reported to exert protective roles in various pathological conditions such as rheumatoid arthritis, chronic obstructive pulmonary disease, cardiovascular and respiratory disorders, and gastrointestinal diseases. Beyond these effects, propolis also displays neuroprotective activity and contributes to immune regulation through its immunomodulatory and immunoinflammatory properties (11, 12).

Given the growing interest in and consumption of kombucha in recent years, the feasibility of producing new types of kombucha using alternative substrates is being investigated (13). Fermented aronia products have been reported to show promise as functional foods with significant health-promoting properties (14). While experiments are being conducted on the use of bee products in kombucha production, the effects of these products (propolis, pollen, etc.) on the bioactive profile and functional potential (diabetic activity and *in vitro* bioaccessibility) of kombucha have been investigated (15, 16). Strategies such as optimizing physicochemical properties, designing new formulations and applying molecular modifications can effectively increase bioavailability (17). Optimizing SCOBY for kombucha, standardizing production processes, and evaluating long-term health effects are among the main issues requiring more comprehensive research in the current literatüre (18).

A literature review revealed that no studies have been conducted on kombucha fermented with aronia tea supplemented with propolis. Beyond addressing this literature gap, the combination of aronia and propolis was selected for its functional and biochemical rationale. Aronia is a rich source of anthocyanins, phenolic acids, and other antioxidant compounds, whereas propolis contains diverse flavonoids, phenolic esters, and other biologically active constituents. Within the acidic, metabolically active kombucha environment, these compounds may undergo partial release, transformation, or increased accessibility during fermentation, potentially influencing the bioactive profile and functional properties of the final beverage. Accordingly, this formulation was designed not as a blind empirical combination, but with the expectation that the complementary phytochemical matrices of aronia and propolis, together with fermentation-associated biochemical changes, may contribute to improved functional potential. Therefore, the main objective of this study was to evaluate the antidiabetic potential and bioaccessibility-related properties of kombucha fermented with aronia tea supplemented with propolis. In addition, the study aimed to develop a health-promoting functional beverage by optimizing the product’s bioactive composition using response surface methodology (RSM). The phenolic compound profile of kombucha samples obtained at different fermentation times was also comparatively evaluated.

## 2 Materials and methods

### 2.1 Preparing kombucha

The following materials were used for kombucha production: “Marcel” brand dried and ground aronia berries and stems (aronia tea) purchased from a local market, powdered sucrose, “Eğriçayır” brand organically certified water-based propolis (33% pure propolis), and kombucha culture (SCOBY) obtained from cultures maintained at the Nutrition and Dietetics Laboratory of Namık Kemal University in Tekirdağ. Kombucha production was carried out at the Nutrition and Dietetics Laboratory of Namık Kemal University in Tekirdağ. Kombucha fermented with aronia tea was coded as “AK,” and kombucha beverage fermented with propolis-enriched aronia tea was coded as “PAK.” Aronia tea was prepared according to the manufacturer’s recommendations. Hundred gram of sucrose was dissolved in 1 liter of boiling water (19). Aronia tea was added to boiling water at concentrations of 6–14 g/L and allowed to infuse for 15 min. The resulting infusion was subsequently filtered through a 0.5 mm filter to remove coarse particulate plant material; this step was applied only for gross particle removal and not for microbiological clarification. Under aseptic conditions, the filtered tea was immediately dispensed into sterile 100 mL glass jars, with 70 mL of medium transferred into each jar. The jars had a mouth diameter of approximately 55 mm and a base diameter of 50 mm, and the same vessel size and filling volume were used throughout all fermentation trials to maintain comparable air–liquid interfacial conditions. The SCOBY used in this study was derived from a previously propagated laboratory culture and was the same inoculum source used in our earlier studies. Although the microbial consortium was not characterized at the species level in the present work, this inoculum was used consistently throughout all fermentation trials to ensure experimental uniformity. The SCOBY was added at 10% (w/v), and pre-produced kombucha tea was incorporated at 100 mL/L as the starter liquid. For the preparation of the PAK samples, the method of Turkoğlu Bacanak and Keyvan (2024) was modified, and water-based propolis (0.50–2.00%) was added to the inoculated tea infusions. The jars were covered with a clean, porous cloth and secured to allow aerobic fermentation while minimizing contamination. Fermentation was carried out in a temperature-controlled dark incubator at 28 ± 1 °C for 14 days. Samples were collected on fermentation days 0, 7, 10, and 14, passed through a 0.5 mm filter to remove coarse suspended material, and stored at −18 ± 1 °C until analysis. All analyses were performed in triplicate.

### 2.2 Response surface methodology (RSM)

The effect of bioactive compounds on the preparation of the propolis-enriched aronia kombucha beverage recipe was analyzed using the response surface method with Minitab Statistical Analysis Software (Minitab 18.1.1). Aronia tea quantity (X_1_) and propolis concentration (X_2_) were determined as independent variables. The amount of aronia tea (X_1_) used in this study (6–14 g/L) was chosen based on both commonly applied kombucha formulations and the need to investigate higher substrate levels for bioactive enrichment (20). Propolis concentration (X_2_) was added to the inoculated tea infusions at a rate of 0.50–2.00%, following the method of Turkoğlu Bacanak and Keyvan (15). Total phenolic content (TPC), total antioxidant capacity (DPPH), and total flavonoid content (TFC) were determined as dependent variables (Table 1). A Central Composite Design was selected as the experimental design, and a 5-level, 2-factor experimental design was created. There are 13 experimental points for optimization. Model adequacy was evaluated by considering the *R*^2^ and adjusted *R*^2^ coefficients, lack-of-fit tests, and ANOVA results.

**Table 1:** Levels of independent variables in the optimization of kombucha beverages fermented with propolis-enhanced aronia tea.

Level	Independent variable	
	Aronia tea Quantity (X_1_)	Propolis Concentration (X_2_)
−1.41	6 g/L	%0.50
−1	8 g/L	%0.87
0	10 g/L	%1.25
1	12 g/L	%1.62
1.41	14 g/L	%2.00

A second-degree polynomial formula was used to determine the relationship between the independent variables (aronia tea amount, propolis concentration) and the responses (TFC, TPC, and DPPH) This polynomial formula is shown in Equation 1:


$$y=\beta_{0}+\Sigma_{i=1}^{3}\beta_{i}X_{i}+\Sigma_{i=1}^{3}\beta_{\mathrm{ii}}X_{i}^{2}+\Sigma_{\begin{array}{c} i=1\\ i<j \end{array}}^{3}\Sigma_{j=1}^{3}\beta_{\mathrm{ij}}X_{i}X_{j}$$ (1)

Formula definition:

Dependent variable (y).

Independent variables (X𝑖 and X𝑗).

Intercept term (β_0_).

Coefficient of the first-order linear equation (β_i_).

Coefficient of the second-order linear equation (β_ii_).

Coefficient of the interaction between the two factors (β_ij_).

### 2.3 Determination of bioactive compounds

#### 2.3.1 Total phenolic compound (TPC) determination

Using a modified version of the Folin–Ciocalteu method described by Singleton and Rossi (1965), total phenolic content (TPC) was determined (21). To determine total phenolic content (TPC), 0.1 mL of each kombucha sample was transferred into a test tube and diluted with 4.5 mL of distilled water. Subsequently, 0.1 mL of Folin–Ciocalteu reagent was added, and the mixture was vortexed for 1 min to ensure complete homogenization. After standing for 3 min, 0.3 mL of 7.5% (w/v) sodium carbonate solution was added. The reaction mixture was vortexed again and then incubated in the dark at room temperature for 2 h. At the end of the incubation period, absorbance was measured at 765 nm using a UV–Vis spectrophotometer (SP-UV/VIS-300SRB), with a reagent blank used as the reference. For digested samples, a sample-specific blank was prepared for each digestion stage, and the endogenous background absorbance of the corresponding matrix was subtracted prior to TPC calculation to account for pH-dependent matrix color changes. Quantification was performed using a calibration curve prepared with gallic acid as the standard, and the results were expressed as μg gallic acid equivalents per mL (μg GAE/mL).

#### 2.3.2 Total flavonoid content (TFC) determination

A modified protocol based on the method of Zhishen et al. was applied to determine the total flavonoid content (TFC) (22). Briefly, 1 mL of each sample was mixed with 4 mL of distilled water, followed by the addition of 0.3 mL of 5% (w/v) sodium nitrite solution. The mixture was allowed to stand at room temperature for 5 min. Subsequently, 0.3 mL of 10% (w/v) aluminum chloride solution was added, and the reaction mixture was gently mixed and allowed to stand for an additional 6 min. Thereafter, 2 mL of 1 M sodium hydroxide was introduced, and the final volume was adjusted to 10 mL with distilled water. Absorbance was measured at 510 nm using a UV–Vis spectrophotometer (SP-UV/VIS-300SRB), with the corresponding blank solution used as the reference. For digested samples, a sample-specific blank was prepared for each digestion stage, and the endogenous background absorbance of the corresponding matrix was subtracted prior to TFC calculation to account for pH-dependent matrix color changes. All measurements were performed in triplicate, and the results were calculated as the mean. Total flavonoid content was expressed as μg catechin equivalents per mL (μg CE/mL).

#### 2.3.3 Determination of total antioxidant capacity

To determine antioxidant capacity, the DPPH (2,2-diphenyl-1-picrylhydrazil) radical scavenging method defined by Brand-Williams et al. (1995) was used, but it was modified to suit the conditions of the study (23). During the analysis, 0.1 mL of the kombucha sample was mixed with 2.9 mL of 0.1 mM ethanol DPPH solution and homogenized using a vortex device. The prepared reaction mixture was incubated in the dark for 30 min to allow the reaction to proceed. Following incubation, absorbance was recorded at 517 nm using a UV–Vis spectrophotometer (SP-UV/VIS-300SRB). The free radical scavenging capacity against DPPH was subsequently calculated according to the equation provided in Equation 2.


$$\text{DPPH}(\%)=[(A_{0}-A_{1})/A_{0}]\;x\;100$$ (2)

A_0_: control absorbance.

A_1_: sample absorbance.

### 2.4 Color analysis

The L*, a*, and b* values of the kombucha samples were measured using a Hunter colorimeter (Color Measuring Device PCE-CSM 5, Germany). Color analysis was performed directly on the unclarified kombucha beverage, without prior filtration or clarification, to preserve the sample’s native structure and reflect the drink’s color characteristics in its directly consumable form. Therefore, the measured L*, a*, and b* values represent the overall optical appearance of the whole beverage matrix. L* is a measure of lightness and darkness ranging from 0 to 100. Zero corresponds to black, and 100 corresponds to white. In the color measurement system, positive (+) values of a* indicate redness, and negative (−) values indicate greenness. Positive (+) values of b* indicate yellowness, while negative (−) values indicate blueness. Chroma (C) and hue angle (h) are expressed by the equations given in Equations 3, 4.


$$C={\left(a^{2}+b^{2}\right)}^{1/2}$$ (3)

$$h=\tan^{-1}(\frac{b}{a})\;$$ (4)

### 2.5 Analysis of phenolic compounds

The polyphenol analysis of kombucha tea was determined using the procedure explained by Portu et al. (2017) involving a chromatographic procedure with an ACE Generix C-18 column (250 × 4.6 mm, 5 μm packing, Agilent) (24). Prior to HPLC-DAD analysis, the kombucha samples were clarified to minimize matrix-related interference and to protect the column from particulate fouling. Accordingly, samples were centrifuged, and the supernatants were filtered through a 0.45 μm membrane filter before injection.

Chromatographic separation was performed using a reversed-phase C18 column with a mobile phase consisting of solvent A (0.1% phosphoric acid in water) and solvent B (acetonitrile), delivered at a flow rate of 0.8 mL/min. The column temperature was maintained at 30 °C, and the injection volume was 10 μL. The gradient elution program was as follows: 0 min, 83% A/17% B; 7 min, 85% A/15% B; 20 min, 80% A/20% B; 24 min, 75% A/25% B; 28 min, 70% A/30% B; 30 min, 60% A/40% B; 32 min, 50% A/50% B; 36 min, 30% A/70% B; and 40 min, 83% A/17% B. Detection was carried out using a diode-array detector (DAD), and chromatograms were monitored at multiple wavelengths (280, 320, and 360 nm) to ensure comprehensive detection of phenolic acids and flavonoids. Phenolic compounds were identified by comparing retention times and UV spectra with those of authentic reference standards. Quantification was performed using external calibration curves constructed with commercially available standards (e.g., Sigma-Aldrich, USA). The calibration curves showed excellent linearity, with coefficients of determination (R^2^) higher than 0.999 for all analyzed compounds. The method validation parameters were also evaluated. The limits of detection (LOD) and limits of quantification (LOQ) were determined based on signal-to-noise ratios of 3 and 10, respectively, and were found to range between 0.01–0.05 μg/mL (LOD) and 0.03–0.15 μg/mL (LOQ), depending on the compound. Recovery values ranged from 92 to 105%, indicating good accuracy and reliability of the analytical method. All analyses were performed in triplicate (n = 3), and the results were expressed as mean values in micrograms per milliliter (μg/mL).

### 2.6 Characterization of kombucha fermented with optimized propolis-enhanced aronia tea

After obtaining the optimized kombucha sample (PAK) and control sample (AK), the bioaccessibility and antidiabetic activity of the bioactive compounds were evaluated.

#### 2.6.1 Bioaccessibility analysis

The kombucha samples were subjected to *in vitro* digestion following a modified protocol based on Minekus et al. (2014). The procedure comprised three consecutive phases: oral digestion using *α*-amylase at pH 7.0, gastric digestion with pepsin at pH 3.0, and intestinal digestion employing pancreatin and fresh bile at pH 7.0. At the end of each simulated digestion phase (oral, gastric, and intestinal), the digested samples were centrifuged, and the resulting supernatants were collected for TPC and TFC analyses. Each treatment was prepared in triplicate to ensure reproducibility (25).

#### 2.6.2 Antidiabetic activity assay

The *in vitro* antidiabetic potential of the kombucha samples was evaluated by measuring their inhibitory activities against *α*-glucosidase and α-amylase, following a modified protocol based on the Worthington Enzyme Manual (Worthington, 1993). Acarbose was used as the positive control in both assays. Prior to analysis, kombucha samples were centrifuged and filtered through a 0.45 μm membrane filter to remove suspended particles, and the clear filtrates were used for enzyme inhibition analyses. All measurements were carried out in triplicate, and absorbance values were recorded using a UV–Vis spectrophotometer (SpectrumInstrument, SP-UV/VIS-300SRB).

For the *α*-amylase inhibition assay, 50 μL of kombucha sample was mixed with 50 μL of α-amylase solution (13 U/mL) prepared in 0.02 M sodium phosphate buffer (pH 6.9) containing 6 mM NaCl. The mixture was pre-incubated at 25 °C for 10 min. Subsequently, 50 μL of 1% (w/v) soluble starch solution prepared in the same buffer was added to initiate the reaction, and the reaction mixture was further incubated at 25 °C for 10 min. The reaction was terminated by adding 100 μL of 3,5-dinitrosalicylic acid (DNS) reagent, followed by heating in a boiling water bath for 5 min. After cooling to room temperature, 1.0 mL of distilled water was added, and the absorbance was measured at 540 nm. A control containing enzyme and substrate without a sample was used to represent 100% enzyme activity, while the corresponding sample blank was prepared to correct for background absorbance.

For the *α*-glucosidase inhibition assay, 50 μL of kombucha sample was mixed with 100 μL of α-glucosidase solution (1.0 U/mL) prepared in 0.1 M phosphate buffer (pH 6.9), and the mixture was pre-incubated at 25 °C for 10 min. Thereafter, 50 μL of 5 mM p-nitrophenyl-α-D-glucopyranoside (pNPG) solution prepared in the same buffer was added as the substrate, and the reaction mixture was incubated at 25 °C for 5 min. The reaction was stopped by adding 2.0 mL of 0.1 M Na2CO3, and the release of p-nitrophenol was measured at 405 nm. A control without a sample and the corresponding reagent blank were included in each run, and acarbose was tested under the same conditions as the reference inhibitor.

The inhibitory activity of the kombucha samples against both enzymes was calculated using the following equation: Inhibition (%) = [1 − (Asample − Asample blank) / (Acontrol − Acontrol blank)] × 100, where Asample is the absorbance of the reaction mixture containing the sample, Asample blank is the absorbance of the corresponding sample blank, Acontrol is the absorbance of the control reaction without inhibitor, and Acontrol blank is the absorbance of the reagent blank. Accordingly, all reported *α*-amylase and α-glucosidase inhibition percentages correspond to assays performed with 50 μL of kombucha filtrate under the fixed reaction conditions described above (26).

### 2.7 Statistical analysis

Statistical analyses were conducted using SPSS 22.0 (SPSS Inc., Chicago, IL, USA). SigmaPlot 12.0 (Systat Software Inc., San Jose, CA, USA) was used for the generation of three-dimensional response surface graphs, while Minitab 18.1.1 was employed for response surface methodology (RSM) analyses. Data are reported as mean ± standard deviation (SD) based on three independent replicates. Before applying parametric tests, the assumptions of normal distribution and homogeneity of variance were assessed using the Shapiro–Wilk and Levene’s tests, respectively. Differences among multiple groups were analyzed by one-way ANOVA, followed by Tukey’s *post hoc* test for pairwise multiple comparisons. As the measurements obtained at different fermentation times originated from separately prepared/sample-collected experimental units rather than repeated observations from the same fermentation vessel, time-point data were treated as independent observations. Accordingly, comparisons between two groups were performed using the Independent Samples t-test, where applicable. Statistical significance was accepted at *p* < 0.05.

## 3 Results and discussion

### 3.1 Optimization of bioactive compounds

RSM was used in the optimization of propolis-enhanced aronia tea kombucha. The dependent variables were determined as TPC, TFC, and DPPH, while the amount of aronia tea (X_1_) and propolis concentration (X_2_) were determined as independent variables. As a result of RSM, the second-degree modeling equation for propolis-enhanced aronia kombucha is shown in Equation 5 for TPC, Equation 6 for TFC, and Equation 7 for DPPH.


$$\begin{array}{l} \mathrm{TPC}(\text{μgGAE}/\mathrm{mL})=226.3+20.41\;X_{1}+2.4\;X_{2}\\ -1.3869\;X_{1}X_{1}-29.50\;X_{2}X_{2}+8.327\;X_{1}X_{2} \end{array}$$ (5)

$$\begin{array}{l} \mathrm{TFC}(\text{μgCE}/\mathrm{mL})=16.52+2.182\;X_{1}+3.13\;X_{2}\\ -0.15119\;X_{1}X_{1}-4.388\;X_{2}X_{2}+0.917\;X_{1}X_{2} \end{array}$$ (6)

$$\begin{array}{l} \text{DPPH}(\%\text{inhibition})=11.20+1.957\;X_{1}-2.73\;X_{2}\\ -0.16821\;X_{1}X_{1}-3.762\;X_{2}X_{2}+1.107\;X_{1}X_{2} \end{array}$$ (7)

Table 2 shows the experimental and RSM results of kombucha tea with varying amounts of aronia tea and propolis concentration for the dependent variables (TPC, TFC, and DPPH). TPC as shown in Table 2, exhibited a positive trend in response to increasing aronia tea amount (X₁) and propolis level (X₂). Experimental values ranged from 319.35 to 358.81 μg GAE/mL, with the lowest TPC value recorded at the minimum aronia tea level (trial 13). TFC ranged from 28.22 to 32.15 μg CE/mL, with increasing trends observed at high X₁ and X₂ levels (Table 2). The lowest TFC values were associated with the minimum aronia tea content, while the highest values occurred in trial 6. DPPH radical scavenging activity exhibited a response range of 15.66–18.67%, with higher antioxidant activity generally paralleling increases in TPC and TFC (Table 2). Overall, increasing the amount of aronia tea had a positive effect on all response variables, while the effect of propolis was supportive at moderate levels and partially limiting at high levels.

**Table 2:** Experimental and RSM data for dependent variables.

Sample	Encoded independent variables		Dependent Variables					
	Aronia tea quantity (X_1_)	Propolis concentration (X_2_)	TPC (μg GAE/mL)		TFC (μg CE/mL)		DPPH (% inhibition)	
			Experimental data	RSM predicted	Experimental data	RSM predicted	Experimental data	RSM predicted
1	14.00	1.25	343.26	342.84	30.56	30.54	15.71	15.71
2	10.00	0.50	327.93	327.17	28.33	28.27	17.22	17.18
3	10.00	1.25	353.27	352.70	31.72	31.74	18.67	18.50
4	8.00	1.63	333.24	335.07	29.47	29.72	15.90	16.11
5	10.00	1.25	353.27	352.70	31.84	31.74	18.46	18.50
6	12.00	1.63	358.81	359.88	32.15	32.31	17.59	17.68
7	12.00	0.88	337.39	338.45	29.82	29.94	16.78	16.82
8	10.00	1.25	353.27	352.70	31.84	31.74	18.46	18.50
9	10.00	1.25	353.46	352.70	31.72	31.74	18.67	18.50
10	8.00	0.88	336.80	338.62	29.89	30.10	18.41	18.57
11	10.00	1.25	353.46	352.70	31.84	31.74	18.46	18.50
12	10.00	2.00	345.87	345.05	30.35	30.27	15.66	15.58
13	6.00	1.25	319.35	318.19	28.22	28.10	16.02	15.90
PAK	11.09	1.42	357.23		32.15		18.18	
Experimental values			346.44		34.15		19.65	
% Difference			3.02%		6.22%		8.08%	

TPC: total phenolic content; TFC: total flavonoid content; DPPH: 1,1-diphenyl-2-picrylhydrazil; GAE: gallic acid equivalent; CE: catechin equivalent; RSM: response surface methodology; PAK: kombucha fermented with optimized propolis-enriched aronia tea.

The maximum optimization values for kombucha fermented with propolis-added aronia tea (PAK) samples are presented in Table 2. According to RSM analysis, the optimal conditions were obtained at 11.09 g/L aronia tea and 1.42% propolis concentration. Under these conditions, the estimated TPC, TFC, and DPPH values were calculated as 357.23 μg GAE/mL, 32.15 μg CE/mL, and 18.18% inhibition, respectively. Under the same conditions, the experimentally obtained TPC, TFC, and DPPH values in PAK samples were determined to be 346.44 μg GAE/mL, 34.15 μg CE/mL, and 19.65% inhibition, respectively. The low differences between experimental and model predictions indicate that the RSM model accurately predicts the response variables in the system.

Synergistic, additive, or antagonistic interactions can occur between propolis-derived compounds and aronia tea polyphenols. High concentrations of these components can lead to antagonistic interactions or a decrease in synergy. The literature clearly states that interactions between polyphenols can shift from synergy to antagonism depending on concentration, and this is particularly common in complex phenolic matrices (27).

**Figure 1:**
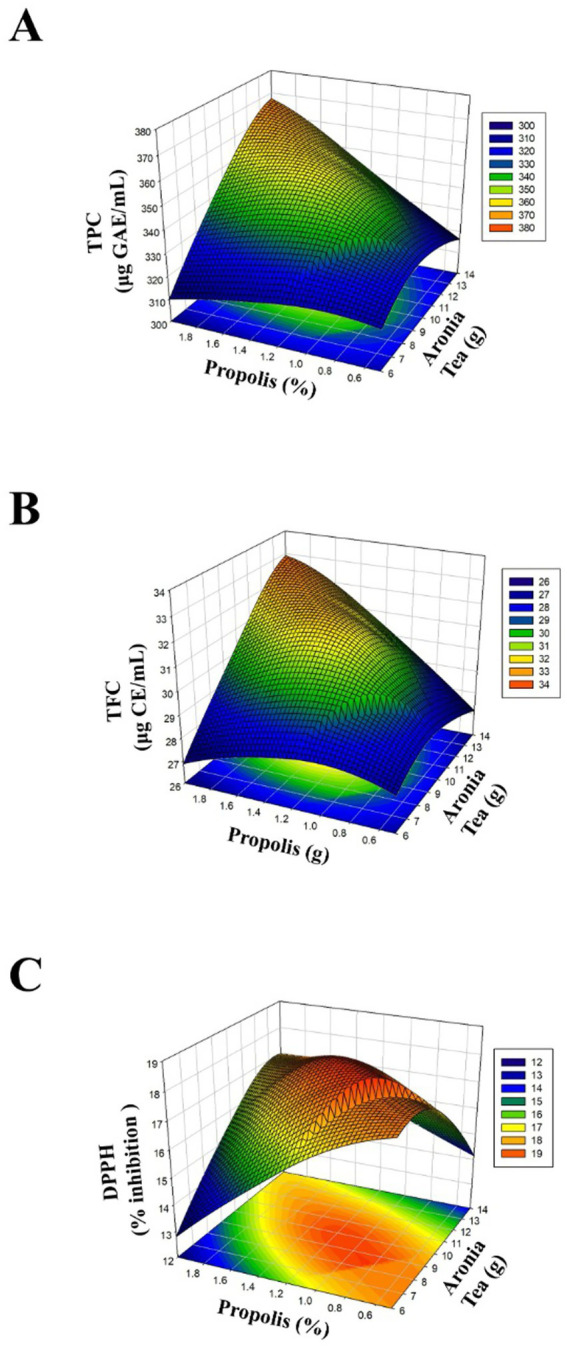
Plots in three dimensions (3D) showing how bioactive compounds behave as a function of significant interaction factors for RSM. **(A)** Total phenolic content (TPC), **(B)** Total flavonoid content (TFC), and **(C)** DPPH radical scavenging activity.

Figure 1 shows that when the combined effects of propolis (%) and aronia tea (g) amounts on TPC, TFC, and DPPH radical scavenging activity are evaluated, the phenolic and flavonoid content and antioxidant capacity increase significantly with increasing amounts of both components. The graphs indicate a significant interaction between the factors and show quadratic effects in the system. In particular, the combined use of medium-high propolis levels with increasing aronia tea amounts creates the region where optimum results are obtained in terms of both phenolic/flavonoid components and antioxidant activity. In contrast, the effect of increasing aronia tea amounts at low propolis levels remains limited. The findings reveal that propolis is a key contributor in terms of phenolic and flavonoid compounds, and these compounds play a decisive role in antioxidant activity.

The ANOVA results presented in Table 3 show that the models established for TPC, TFC, and DPPH responses are statistically significant (*p* = 0.000). High model *F*-values indicate that propolis (X₁) and aronia tea (X₂) are important determinants of the measured responses. While the linear effects of both factors were significant for TPC and TFC, the linear effect of propolis on DPPH was not significant (*p* = 0.330), whereas the effect of aronia tea remained significant. However, the significant quadratic terms (X₁^2^ and X₂^2^) and the binary interaction term (X₁ × X₂) for all responses (*p* = 0.000) reveal a pronounced quadratic behavior and a clear interaction between the factors, indicating the presence of an optimum formulation region. The lack-of-fit test was significant for TPC and TFC but insignificant for DPPH, suggesting that the DPPH model more adequately represented the experimental data. Furthermore, *R*^2^ values above 99%, together with the close agreement between adjusted R^2^ and predicted *R*^2^, indicate high explanatory performance and strong predictive ability of the fitted models. Nevertheless, for TPC and TFC, the significant lack of fit suggests that the predicted optimum conditions should be interpreted with caution as model-based estimates rather than definitive mathematical optima. Moreover, the decline observed at the highest propolis level (2.00%) may be associated with matrix saturation and/or antagonistic interactions between propolis-derived constituents and aronia phenolics under concentrated conditions.

**Table 3:** Analysis of variance (ANOVA) of responses for experiments.

Source	DF	TPC (μg GAE/mL)		TFC (μg CE/mL)		DPPH (% inhibition)	
		*F*-value	*P*-value	*F*-value	*P*-value	*F*-value	*P*-value
Model	5	174.68	0.000	159.12	0.000	150.69	0.000
Linear	2	176.59	0.000	129.98	0.000	41.21	0.000
X_1_	1	231.19	0.000	155.94	0.000	1.10	0.330
X_2_	1	121.99	0.000	104.02	0.000	81.32	0.000
Square	2	220.56	0.000	234.53	0.000	277.65	0.000
X_1_*X_1_	1	357.45	0.000	295.07	0.000	435.69	0.000
X_2_*X_2_	1	199.92	0.000	307.14	0.000	269.41	0.000
2-Way interaction	1	79.08	0.000	66.57	0.000	115.74	0.000
X_1_*X_2_	1	79.08	0.000	66.57	0.000	115.74	0.000
Error	7						
Lack-of-fit	3	423.70	0.000	14.01	0.014	2.87	0.168
Pure error	4						
Total	12						
*R* ^2^		99.20%		99.13%		99.08%	
Adj. *R*^2^		98.64%		98.50%		98.42%	
Pred. *R*^2^		94.43%		94.25%		94.81%	

X_1_: aronia tea; X_2_: propolis; TPC: total phenolic compounds; GAE: gallic acid equivalent; TFC: total flavonoid content; CE: catechin equivalent; DPPH: 1,1-Diphenyl-2-picrylhydrazyl; DF: degrees of freedom; *R*^2^: correlation coefficient; Adj, *R*^2^: Adjusted *R*^2^, Pred, *R*^2^: Predicted *R*^2^; *p* < 0.05: significant differences; *p* < 0.01: highly significant differences.

### 3.2 Determination of bioactive compounds

#### 3.2.1 Total phenolic compounds

It has been reported that phenolic compounds are the main bioactive components in kombucha, and that the type of substrate and fermentation time significantly affect the amount of these compounds (28). In the control group (AK) samples, TPC values were determined to be 328.94 ± 2.52 and 345.28 ± 4.40 μg GAE/mL on days 7 and 14, respectively. The literature reports TPC changes in kombucha produced from different tea types depending on the fermentation period (29). Jakubczyk et al. (2020) reported TPC values of 299.6 ± 3.1 and 320.1 ± 3.5 mg/L on days 7 and 14 in green tea kombucha; 219.5 ± 2.1 and 206.0 ± 1.2 mg/L in black tea; 205.6 ± 3.0 and 228.1 ± 0.5 mg/L in white tea; and 270.5 ± 2.4 and 271.9 ± 3.6 mg/L in red tea. These differences indicate that the chemical composition and biological activity of kombucha vary depending on the type of tea used (30). Another study showed that kombucha beverages prepared from green, oolong, and black teas had reduced phenolic compounds after 3–6 days of fermentation and stabilized after 15 days of fermentation (31).

A significant decrease in TPC values was observed during fermentation in the AK group (*p* < 0.05). In the PAK group, a decrease was found to be significant in the first 7 days (*p* < 0.05), while no statistically significant change was detected in the subsequent periods. Mfopa et al. (2024) recorded the highest phenolic compound content in kombucha after 14 days of fermentation and the lowest content after 28 days of fermentation. Significant differences were reported between days 7 and 35 (32).

In the optimized PAK sample, TPC values were determined to be 347.35 ± 3.94 and 345.28 ± 4.40 μg GAE/mL on days 10 and 14, respectively. Studies in the literature on kombucha with added propolis have reported different TPC values (15). For example, Turkoğlu Bacanak and Keyvan (2024) reported TPC values of 1157.13 and 1941.02 mg GAE/L on the 10th and 14th days of fermentation in black tea kombucha containing 1.5% propolis, respectively. However, it has also been noted that the fermentation process may cause a decrease in the antioxidant profile of propolis (33). Nevertheless, the analysis revealed that TPC values in the PAK sample were statistically significantly higher (*p* < 0.05) compared to the AK sample without propolis. Similarly, Tumbarski et al. (2023) reported that adding 0.02% propolis extract to a functional beverage increased total polyphenols, total flavonoids, and antioxidant activity compared to the control and beverages containing 0.05% potassium sorbate (34) (Table 4).

**Table 4:** TPC results of optimized AK and PAK samples.

Fermentation time (Days)	TPC (μg GAE/mL)	
	AK	PAK
Day 0	334.11 ± 1.99^cA^	367.32 ± 2.56^bB^
Day 7	328.94 ± 2.52^bcA^	352.59 ± 1.89^aB^
Day 10	320.56 ± 1.11^abA^	347.35 ± 3.94^aA^
Day 14	315.42 ± 4.20^aA^	345.28 ± 4.40^aB^

TPC: total phenolic compounds; GAE: gallic acid equivalent; AK: kombucha fermented with aronia tea; PAK: kombucha fermented with optimized propolis-enhanced aronia tea. Results are presented as mean ± standard deviation. Different lowercase letters (a-c) within column values indicate statistical significance (*p* < 0.05). Different uppercase letters (A-B) within row values indicate statistical significance (*p* < 0.05).

#### 3.2.2 Total flavonoid content

The literature reports that changes in the TFC values of kombucha samples have been observed depending on the fermentation period and the type of tea used. In the control group (AK) samples, TFC values on days 0, 7, 10, and 14 of fermentation were determined to be 32.97 ± 0.22, 31.20 ± 0.64, and 30.96 ± 0.13 μg CE/mL, respectively. Indeed, Jakubczyk et al. (2020) found that on the 7th and 14th days of fermentation, green tea kombucha contained 146.8 ± 3.4 and 181.3 ± 4.8 mg/L, respectively; 90.5 ± 0.7 and 126.7 ± 5.2 mg/L in black tea; 83.8 ± 3.3 and 111.6 ± 2.2 mg/L in white tea; and 198.1 ± 2.9 and 242.5 ± 4.8 mg/L in red tea (29). Similarly, Gaggìa et al. (2018) reported variability in TFC levels between days 0–14 of fermentation in green, black, and rooibos tea kombuchas (35). Eroğlu et al. (2024) noted that TFC values in black tea kombucha increased initially during fermentation but decreased in the following days (36).

In this study, when the effect of fermentation time on TFC was evaluated, no statistically significant change was detected in the AK group, whereas a decreasing trend over time was observed in the PAK group. In the intergroup comparison, a significant difference was determined only in the 7-day samples (*p* < 0.05). The findings indicate that the addition of propolis does not generally have a statistically significant effect on TFC levels (Table 5).

**Table 5:** TFC results of optimized AK and PAK samples.

Fermentation time (Days)	TFC (μg CE/mL)	
	AK	PAK
Day 0	32.97 ± 0.22^bA^	39.97 ± 1.22^cA^
Day 7	31.20 ± 0.64^aA^	38.47 ± 0.51^bcB^
Day 10	30.96 ± 0.13^aA^	35.55 ± 0.81^abA^
Day 14	30.32 ± 0.52^aA^	34.33 ± 1.22^aA^

TFC: total flavonoid content; CE: catechin equivalent; AK: kombucha fermented with aronia tea; PAK: kombucha fermented with optimized propolis-enhanced aronia tea. Results are presented as mean ± standard deviation. Different lowercase letters (a-c) within column values indicate statistical significance (*p* < 0.05). Different uppercase letters (A-B) within row values indicate statistical significance (*p* < 0.05).

#### 3.2.3 Total antioxidant capacity

As the fermentation time of kombucha changes, the antioxidant activity measured by DPPH also changes significantly (37). It has been shown that substrate type and microorganism profile also affect kombucha’s antioxidant potential (38). The DPPH value for the control group, AK fermentation, was determined to be 15.94 ± 0.25%, 15.39 ± 0.36%, 15.26 ± 0.53%, and 16.99 ± 0.18% on days 0, 7, 10, and 14, respectively. In the AK group, only the sample from day 14 was found to be statistically different. Although there is no data in the literature on kombucha fermented with aronia tea, it can be compared with different tea samples with similar fermentation periods. Kaewkod et al. (2019) showed the highest DPPH scavenging activity after 15 days of fermentation in green tea kombucha and 9 days of fermentation in oolong tea kombucha in their study. Furthermore, the DPPH scavenging ability of black tea kombucha reached its highest value after the 3rd day of fermentation (31). Cheepchirasuk et al. (2025), in their study, found IC₅₀ values of 4.205 ± 0.357% (h/h) for white tea kombucha, 4.630 ± 0.511% (h/h) for green tea kombucha, and 22.891 ± 1.8% (h/h) for black tea kombucha on the 15th day of fermentation (h/h), green tea kombucha at 4.630 ± 0.511 (h/h), and black tea kombucha at 22.891 ± 1.801 (h/h) on the 15th day of fermentation (39). However, direct comparison between the single-point inhibition percentages obtained in the present study and IC₅₀ values reported in the literature is not methodologically appropriate.

When comparing the groups, a statistically significant difference was observed between the DPPH values of the AK and PAK samples (*p* < 0.05). The results confirm that adding propolis to kombucha significantly increases its radical scavenging capacity. When the relationships among bioactive parameters were evaluated, it was observed that TPC, TFC, and DPPH values exhibited a consistent, positive correlation. In particular, the increase in total phenolic content was strongly associated with increased antioxidant capacity, indicating that phenolic compounds are the primary contributors to radical-scavenging activity in kombucha samples. Moreover, the higher antioxidant capacity observed in PAK samples can be attributed to the synergistic contribution of propolis-derived flavonoids and aronia phenolics. This relationship suggests that the enrichment strategy not only increases individual compound levels but also enhances the beverage’s overall functional potential through combined effects (Table 6).

**Table 6:** DPPH results of optimized AK and PAK samples.

Fermentation time (Days)	DPPH (% inhibition)	
	AK	PAK
Day 0	15.94 ± 0.25^abA^	21.38 ± 0.56^bB^
Day 7	15.39 ± 0.36^aA^	21.16 ± 0.41^abB^
Day 10	15.26 ± 0.53^aA^	20.04 ± 0.17^abB^
Day 14	16.99 ± 0.18^bA^	19.57 ± 0.38^aB^

DPPH: 1,1-Diphenyl-2-picrylhydrazyl; AK: Kombucha fermented with aronia tea; PAK: Kombucha fermented with optimized propolis-enhanced aronia tea. Results are presented as mean ± standard deviation. Different lowercase letters (a-c) within column values indicate statistical significance (*p* < 0.05). Different uppercase letters (A-B) within row values indicate statistical significance (*p* < 0.05).

### 3.3 Color analysis

The chemical changes that occur during the fermentation process affect the final color of the beverage; in particular, the conversion of anthocyanins into derived pigments through reactions with yeast metabolites and flavanols leads to changes in color parameters (40). The color parameter values obtained in this study are shown in Table 7. In this study, the L* values of AK and PAK samples on day 7 were 47.24 ± 0.49 and 35.06 ± 1.13, respectively, and the addition of propolis significantly reduced the L* value (*p* < 0.05). Studies have shown that kombucha has different color parameter values (41). Dartora et al. (2023) reported L* values of 75.12 ± 4.60, 55.90 ± 3.90, and 70.44 ± 4.78 for green, black, and yerba mate tea kombuchas, respectively, after 7 days of fermentation (42).

**Table 7:** Analysis results of L*, a*, b*, C and h° color values during fermentation of kombucha samples.

Color parameter	Fermentation time (Days)	GROUPS	
		AK	PAK
*L*	0	21.78 ± 0.94^aA^	21.71 ± 0.53^aA^
	7	47.24 ± 0.49^dA^	35.06 ± 1.13^dB^
	10	41.95 ± 1.21^cA^	28.95 ± 0.85^cB^
	14	24.73 ± 1.04^bA^	24.82 ± 0.52^bA^
a*	0	4.41 ± 1.08^aA^	2.03 ± 0.57^aB^
	7	5.14 ± 0.11^aA^	10.10 ± 0.27^dB^
	10	5.06 ± 0.21^aA^	4.34 ± 0.49^bA^
	14	7.75 ± 0.73^bA^	8.72 ± 0.57^cA^
b*	0	−1.74 ± 0.50^bA^	−1.90 ± 0.25^aA^
	7	−3.08 ± 0.09^aA^	−0.93 ± 0.04^bB^
	10	1.66 ± 0.07^cA^	−0.54 ± 0.04^bB^
	14	1.26 ± 0.29^cA^	1.20 ± 0.17^cA^
C	0	4.79 ± 0.85^aA^	2.82 ± 0.27^aB^
	7	5.99 ± 0.05^aA^	10.15 ± 0.27^dB^
	10	5.33 ± 0.21^aA^	4.37 ± 0.49^cB^
	14	7.85 ± 0.76^bA^	8.80 ± 0.58^bA^
h*°*	0	337.31 ± 9.78^bA^	316.18 ± 11.19^bA^
	7	329.07 ± 1.29^bA^	354.76 ± 0.08^cB^
	10	18.18 ± 0.98^aA^	352.85 ± 0.57^cB^
	14	9.19 ± 1.46^aA^	7.85 ± 0.61^aA^

AK: Kombucha fermented with aronia tea; PAK: Kombucha fermented with optimized propolis-enhanced aronia tea. Results are presented as mean ± standard deviation. Different lowercase letters (a-d) within column values indicate statistical significance (*p* < 0.05). Different uppercase letters (A-B) within row values indicate statistical significance (*p* < 0.05).

Studies show that kombucha color depends on the substrate (43). In this study, the addition of propolis generally caused significant differences in color parameters on the 7th and 10th days of fermentation (p < 0.05). When the effect of fermentation time was examined, it was determined that L* values, which were similar on day 0, increased on day 7 and the color lightened, but a significant decrease in L* values occurred on days 10 and 14 (*p* < 0.05). Indeed, while Watawana et al. (2018) reported lightening of color in the first days of fermentation, Akarca and Tomar (2020) observed a decrease in L* values during fermentation in different vegetable-based kombuchas (44, 45). Furthermore, Akarca and Tomar (2020) noted that a* values varied during fermentation depending on the product (45).

### 3.4 Phenolic compounds

Secondary plant metabolites are compounds that include phenolic rings, which are linked to one or more hydroxyl groups (46). Table 8 demonstrates that the phenolic profile of AK and PAK samples changed dynamically throughout fermentation, both on a compound-specific and time-dependent basis. In AK, a clear accumulation of certain phenolics over time was observed; for example, rutin increased from 1.18 ± 0.11 μg/mL on day 0 to 3.03 ± 0.11 μg/mL on day 14 (*p* < 0.05). This comparatively lower rutin level in the PAK system may suggest that propolis partially affected SCOBY-mediated hydrolytic biotransformation (47–50). Rutin was found at a relatively high concentration in the phenolic profile of aronia-based kombucha tea. In another study, rutin was similarly reported as the predominant phenolic compound in kombucha prepared with licorice and mint, consistent with our findings. The incorporation of specific plant materials can significantly influence the phytochemical composition of kombucha (51). Similarly, t-ferulic acid increased from 0.66 ± 0.06 to 2.21 ± 0.08 μg/mL in AK (*p* < 0.05), and 4-hydroxybenzoic acid reached levels of 0.28 ± 0.01 μg/mL on day 7 and 1.34 ± 0.05 μg/mL on day 14 (p < 0.05). Compounds that increased with fermentation were also present in the PAK group; for example, t-ferulic acid increased from 1.81 ± 0.25 μg/mL on day 0 to 2.79 ± 0.44 μg/mL on day 14 (*p* < 0.05). In contrast, some compounds were often reported as “n.d.” (e.g., naringin, flavonoids), suggesting that these fractions remained below the detection limit in the product matrix.

**Table 8:** Changes in individual phenolic compounds (μg/mL) in aronia-tea kombucha (AK) and propolis-supplemented aronia-tea kombucha (PAK) during fermentation (0–14 days).

Phenolic compounds (μg/mL)	Fermentation time (Days)	Groups	
		AK	PAK
Chlorogenic acid	0	n.d.	0.00 ± 0.00^a^
	7	0.03 ± 0.00^A^	0.08 ± 0.01^bA^
	10	n.d.	0.00 ± 0.00
	14	n.d.	0.00 ± 0.00
Catechin hydrate	0	0.17 ± 0.01^bA^	0.27 ± 0.04^aA^
	7	0.48 ± 0.03^cA^	0.13 ± 0.01^aB^
	10	0.01 ± 0.00^aA^	0.29 ± 0.02^aB^
	14	0.77 ± 0.03^dA^	0.55 ± 0.08^bA^
Caffeic acid	0	0.14 ± 0.01^bA^	0.46 ± 0.06^bA^
	7	0.08 ± 0.01^aA^	0.13 ± 0.01^aA^
	10	0.18 ± 0.00^cA^	0.21 ± 0.01^aA^
	14	0.31 ± 0.01^dA^	0.24 ± 0.04^aA^
4-Hydroxybenzoic acid	0	0.60 ± 0.06^bA^	1.13 ± 0.16^abA^
	7	0.28 ± 0.01^aA^	0.69 ± 0.04^aB^
	10	0.40 ± 0.01^aA^	1.28 ± 0.11^abA^
	14	1.34 ± 0.05^cA^	1.58 ± 0.25^bA^
*p-*Coumaric acid	0	n.d.	0.00 ± 0.00^a^
	7	n.d.	0.00 ± 0.00^a^
	10	n.d.	0.00 ± 0.00^a^
	14	0.02 ± 0.00^A^	0.02 ± 0.01^bA^
Rutin	0	1.18 ± 0.11^aA^	1.20 ± 0.17^aA^
	7	0.85 ± 0.05^aA^	0.78 ± 0.04^aA^
	10	1.78 ± 0.04^bA^	1.53 ± 0.13^aA^
	14	3.03 ± 0.11^cA^	1.98 ± 0.31^bA^
*Trans*-ferulic acid	0	0.66 ± 0.06^aA^	1.81 ± 0.25^abA^
	7	0.54 ± 0.03^aA^	0.90 ± 0.06^aB^
	10	1.54 ± 0.04^bA^	2.09 ± 0.19^bA^
	14	2.21 ± 0.08^cA^	2.79 ± 0.44^bA^
Hydroxycinnamic acid	0	0.04 ± 0.01^aA^	0.05 ± 0.01^aA^
	7	0.04 ± 0.00^a^	0.07 ± 0.00^b^
	10	0.06 ± 0.00^bA^	0.06 ± 0.01^abA^
	14	0.10 ± 0.00^cA^	0.04 ± 0.01^aB^
Naringin	0	n.d.	n.d.
	7	n.d.	n.d.
	10	n.d.	n.d.
	14	n.d.	n.d.
*o-*Coumaric acid	0	0.00 ± 0.00	0.06 ± 0.01^bA^
	7	n.d.	0.01 ± 0.00^a^
	10	n.d.	0.04 ± 0.00^abB^
	14	0.02 ± 0.00^A^	0.10 ± 0.01^bA^
Rosmarinic acid	0	0.00 ± 0.00^A^	0.85 ± 0.12^aA^
	7	0.07 ± 0.01^A^	0.68 ± 0.04^aB^
	10	n.d.	0.8 ± 0.07^aB^
	14	n.d.	1.11 ± 0.18^aA^
Salicylic acid	0	0.00 ± 0.00	0.35 ± 0.05^abA^
	7	0.00 ± 0.00	0.26 ± 0.01^aB^
	10	0.00 ± 0.00^aA^	0.23 ± 0.02^aB^
	14	0.00 ± 0.00^aA^	0.44 ± 0.06^bA^
Resveratrol	0	0.01 ± 0.00	n.d.
	7	n.d.	n.d.
	10	0.01 ± 0	n.d.
	14	0.03 ± 0	n.d.
Quercetin	0	0.18 ± 0.01^aA^	1.12 ± 0.16^abA^
	7	0.18 ± 0.01^aA^	0.71 ± 0.06^aA^
	10	0.23 ± 0.01^aA^	1.13 ± 0.10^abB^
	14	0.73 ± 0.02^bA^	1.55 ± 0.24^bA^
*Trans*-cinnamic acid	0	0.00 ± 0.00^A^	0.22 ± 0.04^aA^
	7	n.d.	0.37 ± 0.02^abB^
	10	n.d.	0.32 ± 0.03^aB^
	14	n.d.	0.54 ± 0.08^bA^
Naringenin	0	0.00 ± 0.00^aA^	0.28 ± 0.04^aA^
	7	0.02 ± 0.00^bA^	0.37 ± 0.02^abB^
	10	0.00 ± 0.00^aA^	0.39 ± 0.04^abB^
	14	0.07 ± 0.01^cA^	0.47 ± 0.07^bA^
Chrysin	0	0.23 ± 0.02^A^	0.00 ± 0.00
	7	0.85 ± 0.05^A^	17.75 ± 1.08^bB^
	10	1.80 ± 0.05^A^	19.14 ± 1.73^bB^
	14	0.75 ± 0.02^A^	25.34 ± 3.98^bA^
Flavones	0	n.d.	n.d.
	7	n.d.	n.d.
	10	n.d.	n.d.
	14	n.d.	n.d.

AK: Kombucha fermented with aronia tea; PAK: Kombucha fermented with aronia tea supplemented with propolis. The results are presented as the mean ± standard deviation. Different lowercase letters within rows (a–d) indicate statistically significant differences between values (*p* < 0.05). Different uppercase letters within columns (A–B) also indicate statistically significant differences between values (*p* < 0.05). n.d.: not detected.

In the intergroup comparison, the addition of propolis resulted in a marked differentiation in selected phenolic compounds. In particular, chrysin was dramatically higher in the PAK group: on day 7, PAK contained 17.75 ± 1.08 μg/mL, whereas AK contained 0.85 ± 0.05 μg/mL (*p* < 0.05); on day 10, the values remained significantly different at 19.14 ± 1.73 μg/mL for PAK and 1.80 ± 0.05 μg/mL for AK (*p* < 0.05). This pattern supports the notion that propolis makes a substantial contribution to the incorporation and/or preservation of flavonoids such as chrysin in the final product. Phenolic constituents including chrysin and rosmarinic acid are recognized as key markers of the distinctive phenolic composition of propolis, playing a significant role in its antioxidant capacity and associated biological effects (52, 53). These findings reveal that the addition of propolis enriches the phenolic profile of the product. The near-zero Day 0 chrysin response in the PAK sample should not be interpreted as a true absence of this propolis-derived flavonoid, but rather as limited initial recoverability/detectability in the freshly prepared aqueous matrix prior to fermentation-driven matrix transformation However, the marked increase observed in individual compounds such as chrysin should not be interpreted as requiring a proportional increase in spectrophotometrically determined total flavonoid content (TFC), since HPLC quantifies individual flavonoids separately, whereas the Zhishen-based TFC assay provides a broader colorimetric estimate influenced by compound-specific reactivity and matrix effects.

Similarly, quercetin was higher in PAK; on day 0, PAK was 1.12 ± 0.16 μg/mL while AK was 0.18 ± 0.01 μg/mL (*p* < 0.05), and on day 14, PAK was reported as 1.55 ± 0.24 μg/mL and AK as 0.73 ± 0.02 μg/mL (*p* < 0.05). On the other hand, it was also observed that the difference between groups in some parameters was dependent on the time point; For example, while there was a significant difference between AK (0.48 ± 0.03 μg/mL) and PAK (0.13 ± 0.01 μg/mL) for catechin hydrate on day 7 (*p* < 0.05), the values were closer to each other on day 0 (AK 0.17 ± 0.01, PAK 0.27 ± 0.04 μg/mL), and no significant divergence was observed at this point (*p* > 0.05).

### 3.5 Bioaccessibility analysis

Kombucha’s health-promoting potential is said to be enhanced or preserved after gastrointestinal digestion, according to reports (54). It has been noted that the nutritional efficacy of polyphenol-rich foods may be limited due to low bioavailability (55). In line with this, our study examined the polyphenolic compound levels of aronia kombucha (AK) and propolis-enriched aronia kombucha (PAK) during a simulated digestion process (Table 9). The results showed that TPC and TFC values gradually decreased during digestion in both samples. This decrease is thought to be due to the chemical transformations of phenolic compounds. These are by the gut microbiota. It is also due to the release of matrix-bound compounds. These have different stability properties during digestion (56).

**Table 9:** Total phenolic compounds and total flavonoid compounds in AK and PAK end products in the simulated digestion process.

Digestion Stages	TPC (μg GAE/mL)		TFC (μg CE/mL)	
	**AK**	**PAK**	**AK**	**PAK**
Undigested	315.85 ± 2.85^e^	345.19 ± 2.31^e^	30.37 ± 1.2^d^	34.17 ± 1.49^d^
Oral digestion	247.43 ± 3.98^d^	268.93 ± 3.13^d^	23.69 ± 0.94^c^	26.65 ± 1.16^c^
Gastric digestion	167.44 ± 2.26^c^	179.71 ± 2.92^c^	17.77 ± 0.7^b^	19.90 ± 1.01^b^
Intestinal digestion	107.76 ± 1.73^b^	118.34 ± 1.24^b^	12.26 ± 0.49^a^	13.68 ± 0.79^a^
Recovery %	34.11 ± 0.25	34.28 ± 0.29	40.36 ± 0.01	40.03 ± 0.60

TPC: total phenolic compounds; GAE: gallic acid equivalent; TFC: total flavonoid compounds; CE: catechin equivalent; AK: kombucha fermented with aronia tea; PAK: kombucha fermented with optimized propolis-enhanced aronia tea. Results are presented as mean ± standard deviation. Different lowercase letters (a–e) indicate statistically significant differences within the same parameter among comparable values (*p* < 0.05). Recovery (%) values were calculated separately and were not included in the same multiple-comparison grouping as concentration data.

The literature contains different findings regarding the bioavailability and bioaccessibility of kombucha. Q. Zhao et al. (2023) reported that digestion and colonic fermentation could increase antioxidant activity but could have adverse effects on TPC, TFC, and *α*-glucosidase inhibitory activity (57). Değirmencioğlu et al. (2020) reported that the bioavailability of TPC in kombucha produced with different tea types varied depending on the fermentation time and tea type; they found the highest value in oolong tea (58.97%) and the lowest value in black tea (30.63%) (58). In our study, TPC bioaccessibility in AK and PAK samples was 34.11 ± 0.25% and 34.28 ± 0.29%, respectively, and the addition of propolis did not create a statistically significant difference. However, TPC values of PAK samples were significantly higher than those of AK samples in all digestion stages (*p* < 0.05).

In terms of total flavonoid bioavailability, Liang et al. (2024) reported values of 8.93% in unfermented green tea and 76.06% in green tea kombucha (59). In this study, TFC bioaccessibility in AK and PAK samples was determined to be 40.36 ± 0.01% and 40.03 ± 0.60%, respectively; no significant effect of propolis addition on bioaccessibility was found. However, it was determined that the TFC value of the PAK sample was significantly higher than that of AK before digestion (*p* < 0.05).

### 3.6 Determination of antidiabetic activity

Inhibiting the *α*-glucosidase enzyme is an effective way of controlling hyperglycaemia, and this mechanism is recognized as an important strategy for treating type 2 diabetes (60). Although it has been reported that *α*-glucosidase inhibitory activity is associated with total phenolic and flavonoid content, further studies are needed to identify the key compounds responsible (61). In the study, the *α*-glucosidase inhibition values of AK and PAK samples were determined to be 34.65 ± 0.94% and 42.04 ± 0.42%, respectively, and it was found that the addition of propolis significantly increased the inhibitory effect (*p* < 0.05) (Table 10). A similar study reported that green tea and kombucha showed a significant inhibitory effect on *α*-glucosidase, but green tea had higher activity (78.17%; 73.64%) (62). It has been reported that 3-caffeoylquinic acid and 4-caffeoylquinic acid isolated from aronia juice are α-glucosidase inhibitors, and it is thought that the effect in AK and PAK samples may be related to these compounds (63).

**Table 10:** α-Glucosidase and α-Amylase inhibitory activities of AK and PAK end products.

α-Glucosidase Inhibitory activity (%)		α-Amylase Inhibitory activity (%)	
AK	PAK	AK	PAK
34.65 ± 0.94A	42.04 ± 0.42B	38.34 ± 0.63A	44.68 ± 1.47B

AK: Kombucha fermented with aronia tea; PAK: Kombucha fermented with aronia tea supplemented with propolis. Results are presented as mean ± standard deviation. Different capital letters within row values (A-B) indicate statistical significance between values (*p* < 0.05).

It has also been noted that kombucha can inhibit α-amylase activity, and this effect may increase during the fermentation process (64). In the study, α-amylase inhibition in AK and PAK samples was found to be 38.34 ± 0.63% and 44.68 ± 1.47%, respectively; the addition of propolis was found to significantly increase this activity (*p* < 0.05). Compared to black tea, kombucha was reported to suppress *α*-amylase activity in plasma and pancreas more strongly and reduce blood glucose levels more effectively (65). Oolong and yellow tea kombuchas have been reported to exhibit high inhibitory effects on both α-amylase and α-glucosidase; specifically, α-amylase activity increased with fermentation, while α-glucosidase inhibition remained at a high level (66). It is suggested that pH changes and the biotransformation of tea compounds may play a role in this mechanism (67). Furthermore, it has been reported that the hypoglycaemic effect of kombucha may not be limited to enzyme inhibition alone but may also occur through the modulation of the gut microbiota (68). The enzyme inhibition data obtained in the present study should be interpreted as comparative *in vitro* screening results under fixed assay conditions, with acarbose serving as the reference inhibitor. The observed increase in α-glucosidase and α-amylase inhibition in PAK samples is closely associated with the elevated phenolic and flavonoid content. Correlation analysis indicates that bioactive compounds, particularly flavonoids such as chrysin and quercetin, may play a key role in enzyme inhibition mechanisms. These findings support the hypothesis that phenolic enrichment enhances not only antioxidant properties but also antidiabetic potential through enzyme-targeted interactions.

### 3.7 Multivariate assessment of AK and PAK kombucha: PCA and Pearson correlation analysis

**Figure 2:**
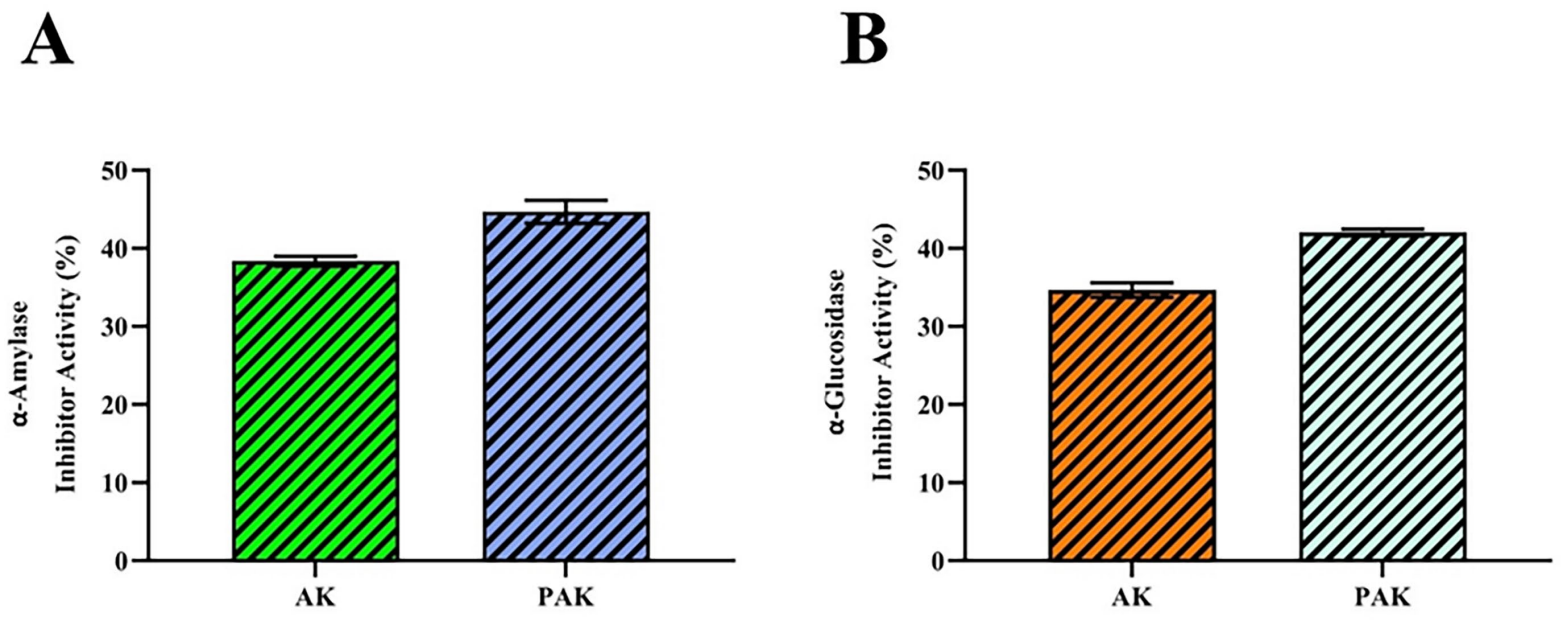
Antidiabetic inhibitory activities **(A,B)** of kombucha fermented with aronia tea (AK) and kombucha fermented with optimized propolis-enhanced aronia tea and (PAK).

**Figure 3:**
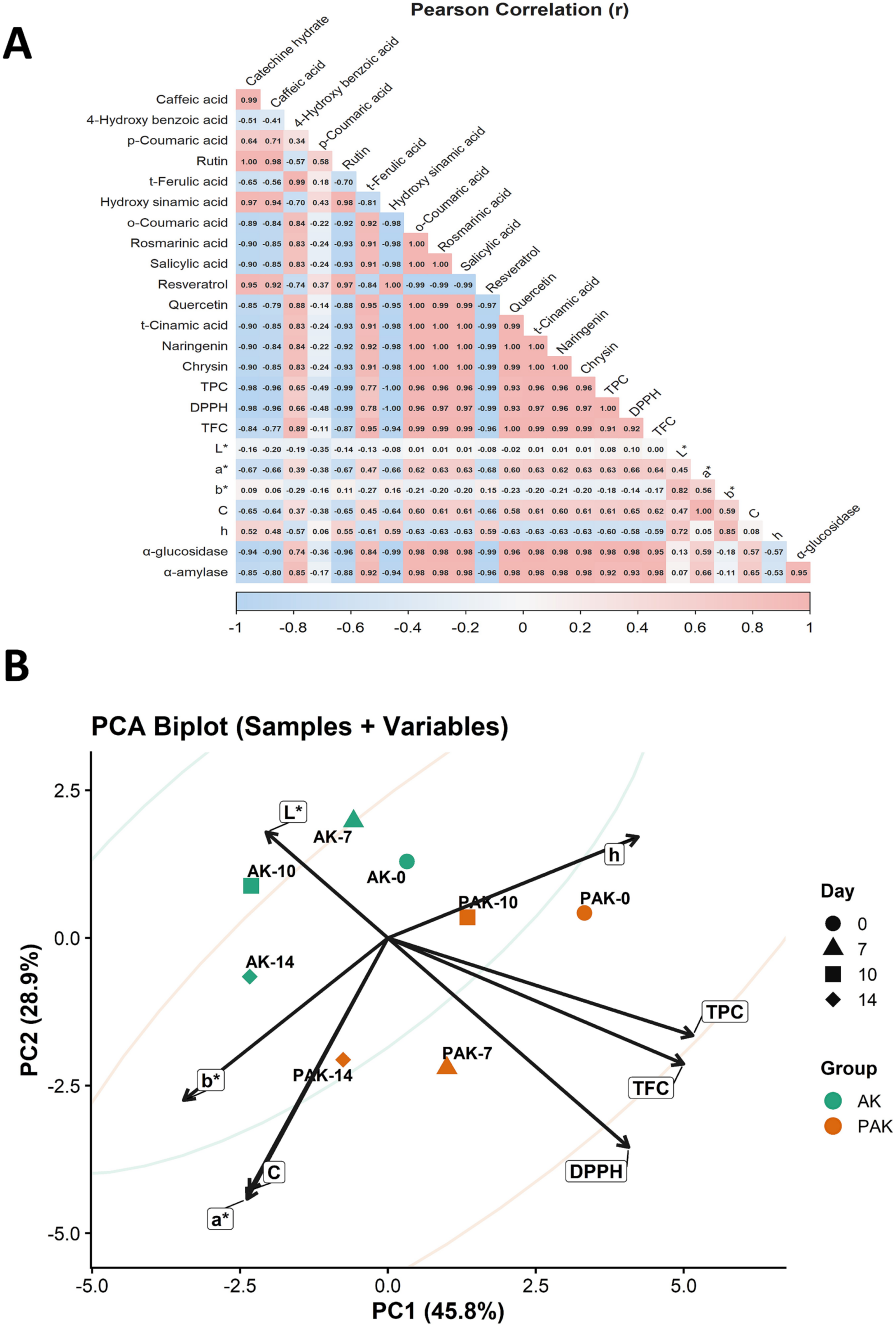
Integrated multivariate statistical evaluation of AK (aronia tea–fermented kombucha) and PAK (aronia tea–fermented kombucha fortified with optimized propolis). **(A)** Pearson correlation heatmap (*r*) with two-tailed significance levels (*p* < 0.05, *p < 0.01, **p < 0.001; n.s., not significant). **(B)** PCA biplot/score plot based on standardized variables (*z*-scores), illustrating sample distribution together with variable loadings (explained variance of PC1 and PC2 is indicated on the axes).

The Pearson correlation heat map given in Figures 2, 3A shows that the relationships between phenolic profile–antioxidant capacity–color parameters–enzyme inhibition in aronia kombucha exhibit a highly distinct structure. Particularly noteworthy are very high positive correlations between some phenolics, such as caffeic acid–catechin hydrate (*r* = 0.99) and rutin–caffeic acid (*r* = 1.00). In contrast, resveratrol showed a strong inverse relationship with rosmarinic/salicylic acid (*r* = −0.99); however, correlations involving compounds with predominantly non-detectable or near-constant values should be interpreted with caution. In functional output variables, antioxidant capacity indicators strongly correlate with each other and with total phenolics (TPC–DPPH *r* = 1.00, TPC–TFC *r* = 0.96, DPPH–TFC *r* = 0.92). Enzyme inhibition metrics also follow this axis; Inhibition of *α*-glucosidase and α-amylase showed a high positive correlation with TPC/DPPH/TFC (e.g., α-glucosidase–DPPH *r* = 0.98, α-amylase–TPC *r* = 0.98), while inverse relationships were particularly prominent with h (hue angle) (α-glucosidase–h *r* = −0.57, α-amylase–h *r* = −0.53). Among the color parameters, the moderate-to-high positive correlation of α* with the bioactive/antioxidant axis (e.g., α*–DPPH *r* = 0.66, α*–TPC *r* = 0.60) supports the idea that the shift in product color may be synchronous with phenolic/antioxidant dynamics.

The PCA biplot in Figure 3B clearly reveals the trajectory of the samples in a multivariate space over time (days 0–7–10–14) in addition to the “group effect.” The first two components explain 74.7% of the total variance (PC1 = 45.8%, PC2 = 28.9%), indicating that the main pattern of time-dependent variation in AK and PAK can be reliably interpreted in this plane. Vector directions show that TPC/TFC/DPPH move together in the positive direction of PC1; L* and, to some extent, h are positioned more in the upper region (PC2 positive); and a*, C, and b* are directed toward the lower-left region. Within this framework, the displacement of the samples over time is evident: AK samples generally shift toward the negative of PC1 as time progresses from day 0, and on day 14 they approach the lower-left region (AK-14), becoming more closely tied to the axis associated with color saturation/hue parameters. In the PAK samples, the initial (PAK-0) sample was closer to the h vector/positive region, while the day 7 sample (PAK-7) exhibited a position closer to the region carrying the effect of TPC/TFC/DPPH vectors, suggesting that propolis optimization more clearly correlated with the bioactive-antioxidant axis in the mid-fermentation phase; the shift of PAK-14 to the lower-left region later on points to a late-stage profile where color parameters (especially a*, C) drive the separation more. This time-based PCA pattern, consistent with the strong TPC–DPPH–TFC association (*r* = 0.92–1.00 range) and high correlations with enzyme inhibition (e.g., *r* ≈ 0.96–0.98) in Figure 3A, shows that the balance between the “functional/bioactive axis” and the “color axis” determined the position of the samples as fermentation progressed. Overall, the integration of correlation analysis and PCA clearly demonstrates that the functional properties of kombucha are primarily governed by the phenolic composition and its dynamic transformation during fermentation. The strong clustering of TPC, TFC, DPPH, and enzyme inhibition variables along the same axis confirms their interdependent behavior. This multivariate structure highlights that improvements in antioxidant capacity are directly reflected in enhanced biological activity, particularly antidiabetic potential. Therefore, the functional performance of the developed kombucha formulations can be attributed to a coordinated bioactive network rather than isolated compound effects.

## 4 Conclusion

This study demonstrates that enriching aronia tea-based kombucha with propolis (PAK) enhances its bioactive composition, phenolic profile, and antidiabetic potential. The RSM approach indicated that the amount of aronia tea (X_1_) and the concentration of propolis (X_2_) were significant factors influencing TPC, TFC, and DPPH values, and the optimum formulation was determined as 11.09 g/L aronia tea and 1.42% propolis. During fermentation, PAK samples exhibited higher total phenolic content than the control group, along with a more diverse phenolic compound profile. Fermentation-related increases were particularly evident in compounds such as rutin and trans-ferulic acid, while the addition of propolis contributed to elevated levels of flavonoids such as chrysin and quercetin, supporting the beverage’s functional enrichment. Under simulated gastrointestinal digestion conditions, a decrease in phenolic and flavonoid contents was observed in all samples. Although PAK samples generally retained higher levels than the control (AK) throughout the digestion phases, these differences were not statistically significant. Notably, PAK exhibited stronger *α*-glucosidase and α-amylase inhibitory activities, suggesting that phenolic enrichment may contribute to the product’s antidiabetic potential. However, these findings should be interpreted with caution and supported by further *in vivo* and clinical studies. In addition, safety-related considerations should be taken into account, since propolis-containing formulations may not be suitable for all consumers, particularly individuals with hypersensitivity to bee products or pollen-related allergens. Therefore, the present formulation should be regarded as a promising functional beverage candidate rather than a universally applicable product. Future research should focus on shelf life, sensory properties, safety evaluation, interactions with the microbiota, and clinical validation to better understand the functional implications of propolis-enriched aronia kombucha.

## Data Availability

The raw data supporting the conclusions of this article will be made available by the authors, without undue reservation.

## References

[1] de Melo LM Soares MG Bevilaqua GC Schmidt VCR de Lima M. Historical overview and current perspectives on kombucha and SCOBY: a literature review and bibliometrics. Food Biosci. (2024) 59:104081. doi: 10.1016/J.FBIO.2024.104081

[2] Huang R. Exploring kombucha: production, microbiota biotransformation, flavor, health benefits and potential risks. Cite this. ACS Food Sci Technol. (2024) 4.7:1610–1625. doi: 10.1021/acsfoodscitech.4c00242

[3] Andrade DKA Wang B Lima EMF Shebeko SK Ermakov AM Khramova VN . Kombucha: an old tradition into a new concept of a beneficial, health-promoting beverage. Foods. (2025) 14:1547. doi: 10.3390/FOODS14091547 40361629 PMC12071948

[4] Mohd Ariff R Chai XY Chang LS Fazry S Othman BA Babji AS . Recent trends in kombucha: conventional and alternative fermentation in development of novel beverage. Food Biosci. (2023) 53:102714. doi: 10.1016/J.FBIO.2023.102714

[5] Prajapati K Prajapati J Patel D Patel R Varshnei A Saraf M . Multidisciplinary advances in kombucha fermentation, health efficacy, and market evolution. Arch Microbiol. (2024) 206:366. doi: 10.1007/S00203-024-04086-1, 39098983

[6] Jayabalan R Waisundara VY. "Kombucha as a functional beverage". In: Functional and Medicinal Beverages: Volume 11: The Science of Beverages (2019). p. 413–46.

[7] Aung T Kim MJ. A comprehensive review on kombucha biofilms: a promising candidate for sustainable food product development. Trends Food Sci Technol. (2024) 144:104325. doi: 10.1016/J.TIFS.2024.104325

[8] Duman H Üner B Sarıtaş S Bolat E Yalçıntaş YM Kalkan AE . Exploring the potential of black chokeberry (*Aronia melanocarpa*) as a health-enhancing agent: a comprehensive overview. J Food Biochem. (2025) 2025:8899523. doi: 10.1155/JFBC/8899523

[9] Go MY Kim J Jeon CY Shin DW. Functional activities and mechanisms of *Aronia melanocarpa* in our health. Curr Issues Mol Biol. (2024) 46:8071–87. doi: 10.3390/CIMB46080477, 39194694 PMC11352306

[10] Ren Y Frank T Meyer G Lei J Grebenc JR Slaughter R . Potential benefits of black chokeberry (*Aronia melanocarpa*) fruits and their constituents in improving human health. Molecules. (2022) 27:27. doi: 10.3390/MOLECULES27227823, 36431924 PMC9696386

[11] Felício IM Cavalcanti AMT Baranger K de Oliveira Junior RG Poirot B Picot L . Brazilian propolis: chemical composition, regional variability, and bioactive potential. Fitoterapia. (2025) 185:106687. doi: 10.1016/J.FITOTE.2025.106687, 40532983

[12] Zullkiflee N Taha H Usman A. Propolis: its role and efficacy in human health and diseases. Molecules. (2022) 27:27. doi: 10.3390/MOLECULES27186120, 36144852 PMC9504311

[13] Freitas A Sousa P Wurlitzer N. Alternative raw materials in kombucha production. Int J Gastron Food Sci. (2022) 30:100594. doi: 10.1016/J.IJGFS.2022.100594

[14] Frumuzachi O Rohn S Mocan A. Fermented black chokeberry (*Aronia melanocarpa* (Michx.) Elliott) products – a systematic review on the composition and current scientific evidence of possible health benefits. Food Res Int. (2024) 196:115094. doi: 10.1016/J.FOODRES.2024.115094, 39614570

[15] Turkoğlu Bacanak R Keyvan E. Assessment of propolis-fermented Kombucha tea’s microbiological, physicochemical and sensory characteristics. Emir J Food Agric. (2024) 36:1–7. doi: 10.3897/EJFA.2024.118977

[16] Türkol M Maviş ÇY Yıkmış S. From chemistry to functionality: HPLC–DAD/LC–MS/MS characterization of bee product-enriched *Prunus spinosa* L. kombucha with in vitro antidiabetic activity and bioaccessibility. ACS Omega. (2026) 11:14048–63. doi: 10.1021/acsomega.6c00008, 41799090 PMC12961545

[17] Xie L Hong Y Hu Y Li H Lou J Zhou X. Prioritizing oral bioavailability in drug development strategies. Future Med Chem. (2025) 17:149–51. doi: 10.1080/17568919.2024.2444871;PAGE:STRING:ARTICLE/CHAPTER, 39723437 PMC11749343

[18] Alkan G Konar Erol NM Yıkmış S Er H Öğüt S Yinanç A. Kombucha’s functional features and fermentation dynamics: a bibliometric assessment in sustainable food production. Front Sustain Food Syst. (2025) 9:1593348. doi: 10.3389/fsufs.2025.1593348

[19] Xiong RG Zhou DD Cheng J Wu SX Saimaiti A Huang SY . Preparation and evaluation of liquorice (*Glycyrrhiza uralensis*) and ginger (*Zingiber officinale*) kombucha beverage based on antioxidant capacities, phenolic compounds and sensory qualities. Int J Gastron Food Sci. (2024) 35:100869. doi: 10.1016/J.IJGFS.2024.100869

[20] Kilmanoglu H Yigit Cinar A Durak MZ. Evaluation of microbiota-induced changes in biochemical, sensory properties and volatile profile of kombucha produced by reformed microbial community. Food Chem X. (2024) 22:101469. doi: 10.1016/J.FOCHX.2024.101469, 38808165 PMC11130685

[21] Singleton VL Rossi JA. Colorimetry of total phenolics with phosphomolybdic-phosphotungstic acid reagents. Am J Enol Vitic. (1965) 16:144–58. doi: 10.5344/AJEV.1965.16.3.144

[22] Zhishen J Mengcheng T Jianming W. The determination of flavonoid contents in mulberry and their scavenging effects on superoxide radicals. Food Chem. (1999) 64:555–9. doi: 10.1016/S0308-8146(98)00102-2

[23] Brand-Williams W Cuvelier ME Berset C. Use of a free radical method to evaluate antioxidant activity. LWT-Food Sci Technol. (1995) 28:25–30. doi: 10.1016/S0023-6438(95)80008-5

[24] Portu J López R Santamaría P Garde-Cerdán T. Elicitation with methyl jasmonate supported by precursor feeding with phenylalanine: effect on Garnacha grape phenolic content. Food Chem. (2017) 237:416–22. doi: 10.1016/J.FOODCHEM.2017.05.126, 28764015

[25] Minekus M Alminger M Alvito P Ballance S Bohn T Bourlieu C . A standardised static in vitro digestion method suitable for food - an international consensus. Food Funct. (2014) 5:1113–24. doi: 10.1039/C3FO60702J, 24803111

[26] Worthington V. Worthington Enzyme Manual. Enzymes and Related Biochemicals. Lakewood, NJ: Wohington Biochemical Company (1993). p. 36–41.

[27] Wang S Meckling KA Marcone MF Kakuda Y Tsao R. Synergistic, additive, and antagonistic effects of food mixtures on total antioxidant capacities. J Agric Food Chem. (2011) 59:960–8. doi: 10.1021/jf1040977, 21222468

[28] Bortolomedi BM Paglarini CS Brod FCA. Bioactive compounds in kombucha: a review of substrate effect and fermentation conditions. Food Chem. (2022) 385:132719. doi: 10.1016/J.FOODCHEM.2022.132719, 35318172

[29] Jakubczyk K Kałduńska J Kochman J Janda K. Chemical profile and antioxidant activity of the Kombucha beverage derived from white, green, black and red tea. Antioxidants. (2020) 9:447. doi: 10.3390/ANTIOX9050447 32455926 PMC7278673

[30] Mihai RA Cubi-Insuaste NS Catana RD. Biological activity and phenolic content of kombucha beverages under the influence of different tea extract substrates. Fermentation. (2024) 10:338. doi: 10.3390/FERMENTATION10070338

[31] Kaewkod T Bovonsombut S Tragoolpua Y. Efficacy of kombucha obtained from green, oolong, and black teas on inhibition of pathogenic bacteria, antioxidation, and toxicity on colorectal cancer cell line. Microorganisms. (2019) 7:700. doi: 10.3390/MICROORGANISMS7120700 31847423 PMC6956236

[32] Mfopa AN Kemzeu R Fokom R Yamthe LRT Dize D Boyom FF. Phenolic compounds, antioxidant and antileishmanial activities of kombucha as affected by fermentation time. Heliyon. (2024) 10:e40463. doi: 10.1016/J.HELIYON.2024.E40463, 39641030 PMC11617884

[33] Garzarella EU Navajas-Porras B Pérez-Burillo S Ullah H Esposito C Santarcangelo C . Evaluating the effects of a standardized polyphenol mixture extracted from poplar-type propolis on healthy and diseased human gut microbiota. Biomed Pharmacother. (2022) 148:112759. doi: 10.1016/J.BIOPHA.2022.112759, 35248845

[34] Tumbarski Y Petrova N Ivanov I Petkova N Ivanova P Yanakieva V . Assessment of the bioactivity, preservation potential and sensory acceptance of a propolis extract applied in a functional fruit-herbal beverage. Food Sci Appl Biotechnol. (2023) 6:320–30. doi: 10.30721/FSAB2023.V6.I2.302

[35] Gaggìa F Baffoni L Galiano M Nielsen DS Jakobsen RR Castro-Mejía JL . Kombucha beverage from green, black and rooibos teas: a comparative study looking at microbiology, chemistry and antioxidant activity. Nutrients. (2019) 11:1. doi: 10.3390/NU11010001 PMC6356548 30577416

[36] Eroğlu B Deli̇k E Emine TEFON-ÖZTÜRK B. Development of an alternative kombucha drink from gilaburu juice: Gilaburu-flavoured kombucha. Nat Sci. (2024) 42:805–13. doi: 10.14744/sigma.2024.00000

[37] Kartika DA Leswana NF Taufiqurrahman M. Antioxidant activity in kombucha (Scooby) tea based on fermentation duration with DPPH (1,1-diphenyl-2-picrylhydrazyl) method. Jurnal Sains dan Teknologi Farmasi Indonesia. (2025) 14:84–90. doi: 10.58327/JSTFI.V14I1.89

[38] Kim H Hur S Lim J Jin K Yang T h Keehm I s . Enhancement of the phenolic compounds and antioxidant activities of kombucha prepared using specific bacterial and yeast. Food Biosci. (2023) 56:103431. doi: 10.1016/j.fbio.2023.103431

[39] Cheepchirasuk N Kaewkod T Suriyaprom S Intachaisri V Ngamsaard P Tragoolpua Y. Functional metabolites and inhibitory efficacy of kombucha beverage on pathogenic bacteria, free radicals and inflammation. Sci Rep. (2025) 15:19187. doi: 10.1038/s41598-025-03545-z 40451934 PMC12127459

[40] Leborgne C Ducasse MA Meudec E Carrillo S Verbaere A Sommerer N . Multi-method study of the impact of fermentation on the polyphenol composition and color of Grenache, Cinsault, and Syrah rosé wines. Food Chem. (2023) 403:134396. doi: 10.1016/J.FOODCHEM.2022.134396, 36358071

[41] Santos L d M Roselino MN Alves J d C Viana SNA dos Reis Requião E Santos Ferro JMRB . Production and characterization of kombucha-like beverage by cocoa (*Theobroma cacao*) by-product as raw material. Fut Foods. (2025) 11:100528. doi: 10.1016/j.fufo.2024.100528

[42] Dartora B Crepalde LT Hickert LR Fabricio MF Ayub MAZ Veras FF . Kombuchas from black tea, green tea, and yerba-mate decocts: perceived sensory map, emotions, and physicochemical parameters. Int J Gastron Food Sci. (2023) 33:100789. doi: 10.1016/J.IJGFS.2023.100789

[43] Yıkmış S Tuğgüm S. Evaluation of microbiological, physicochemical and sensorial properties of purple basil kombucha beverage. Turk J Agric Food Sci Technol. (2019) 7:1321–7. doi: 10.24925/TURJAF.V7I9.1321-1327.2550

[44] Watawana MI Jayawardena N Waisundara VY. Value-added tea (*Camellia sinesis*) as a functional food using the kombucha “tea fungus.”. Chiang Mai J Sci. (2018) 45:136–46.

[45] Akarca G Tomar O. Kırmızı ve mor sebzelerle hazırlanan kombucha çaylarının kalite özelliklerinin belirlenmesi. Mediterr Agric Sci. (2020) 33:215–22. doi: 10.29136/MEDITERRANEAN.680360

[46] Yusoff IM Mat Taher Z Rahmat Z Chua LS. A review of ultrasound-assisted extraction for plant bioactive compounds: Phenolics, flavonoids, thymols, saponins and proteins. Food Res Int. (2022) 157:111268. doi: 10.1016/j.foodres.2022.111268, 35761580

[47] Lu C Zhang J Zhao X Zi Y Xiao X. Biotransformation of phenolic acids in foods: pathways, key enzymes, and technological applications. Foods. (2025) 14:2187. doi: 10.3390/foods14132187 40646939 PMC12248444

[48] Andreson M Kazantseva J Kuldjärv R Malv E Vaikma H Kaleda A . Dynamics of microbial communities, flavor, and physicochemical properties of kombucha-fermented *Sargassum fusiforme* beverage during fermentation. LWT. (2024) 192:115729. doi: 10.1016/j.ijfoodmicro.2022.109715, 35567890

[49] Oršolić N. Allergic inflammation: effect of Propolis and its flavonoids. Molecules. (2022) 27:27. doi: 10.3390/molecules27196694, 36235230 PMC9570745

[50] Silva-Carvalho R Baltazar F Almeida-Aguiar C. Propolis: a complex natural product with a plethora of biological activities that can be explored for drug development. Evid Based Complement Alternat Med. (2015) 2015:1–29. doi: 10.1155/2015/206439, 26106433 PMC4461776

[51] Jakubczyk K Melkis K Maciejewska-Markiewicz D Muzykiewicz-Szymańska A Nowak A Skonieczna-Żydecka K. Innovative approaches to enhancing kombucha through flavour additives: a phytochemical and antioxidant analysis. Food Funct. (2025) 16:1442–57. doi: 10.1039/d4fo05135a, 39898619

[52] Fidan M İnal B Tokgün O Çelikkaya B Teğin İ Yabalak E. Propolis as a functional plant-derived food: antioxidant and anti-cancer properties from Şırnak and Hakkari regions. Eur Food Res Technol. (2025) 251:2945–58. doi: 10.1007/s00217-025-04806-x

[53] Birinci C Kolayli S. A comparative study of phenolic and antioxidant properties of propolis and sumac (*Rhus coriaria* L.). Uludag Aricilik Dergisi. (2025) 25:131–9. doi: 10.31467/uluaricilik.1624649

[54] Ghion G Sica J Massaro S Tarrah A Devold TG Porcellato D . Functional compound bioaccessibility and microbial viability in green and black tea kombucha during simulated digestion. Foods. (2025) 14:2770. doi: 10.3390/FOODS14162770/S1 40870682 PMC12385410

[55] Czubinski J Wroblewska K Czyzniejewski M Górnaś P Kachlicki P Siger A. Bioaccessibility of defatted lupin seed phenolic compounds in a standardized static *in vitro* digestion system. Food Res Int. (2019) 116:1126–34. doi: 10.1016/J.FOODRES.2018.09.057, 30716897

[56] Odriozola-Serrano I Nogueira DP Esparza I Vaz AA Jiménez-Moreno N Martín-Belloso O . Stability and bioaccessibility of phenolic compounds in rosehip extracts during in vitro digestion. Antioxidants. (2023) 12:1035. doi: 10.3390/ANTIOX12051035, 37237901 PMC10215894

[57] Zhao Q Wang Z Wang X Yan X Guo Q Yue Y . The bioaccessibility, bioavailability, bioactivity, and prebiotic effects of phenolic compounds from raw and solid-fermented mulberry leaves during in vitro digestion and colonic fermentation. Food Res Int. (2023) 165:112493. doi: 10.1016/J.FOODRES.2023.112493, 36869449

[58] Değirmencioğlu N Yıldız E Sahan Y Güldas M Gürbüz O. Impact of tea leaves types on antioxidant properties and bioaccessibility of kombucha. J Food Sci Technol. (2020) 58:2304–12. doi: 10.1007/S13197-020-04741-7, 33967327 PMC8076432

[59] Liang W Wang X Zhang L Jiao S Song H Sun J . Changes and biotransformation mechanism of main functional compounds during kombucha fermentation by the pure cultured tea fungus. Food Chem. (2024) 458:140242. doi: 10.1016/J.FOODCHEM.2024.140242, 38943965

[60] Zhang R Zhang Y Huang G Xin X Tang L Li H . Chemical synthesis, inhibitory activity and molecular mechanism of 1-deoxynojirimycin-chrysin as a potent α-glucosidase inhibitor. RSC Adv. (2021) 11:38703–11. doi: 10.1039/D1RA07753H, 35493254 PMC9044198

[61] Tu J Liu G Jin Y Tang C Yao T Zhuo J . Enrichment of γ-aminobutyric acid in mulberry leaves and the inhibitory effects of the water extract on ACE and α-glucosidase activity. Ind Crop Prod. (2022) 177:114485. doi: 10.1016/J.INDCROP.2021.114485

[62] Teixeira Oliveira J da Machado Costa F Gonçalvez Silva T Dotto Simões G dos Santos Pereira E da Quevedo Costa P . Green tea and kombucha characterization: phenolic composition, antioxidant capacity and enzymatic inhibition potential. Food Chem. (2023) 408:135206. doi: 10.1016/J.FOODCHEM.2022.135206, 36528993

[63] Imai M Yamane T Kozuka M Takenaka S Sakamoto T Ishida T . Caffeoylquinic acids from aronia juice inhibit both dipeptidyl peptidase IV and α-glucosidase activities. LWT. (2020) 129:109544. doi: 10.1016/J.LWT.2020.109544

[64] Kallel L Desseaux V Hamdi M Stocker P Hassan Ajandouz E. Insights into the fermentation biochemistry of Kombucha teas and potential impacts of Kombucha drinking on starch digestion. Food Res Int. (2012) 49:226–32. doi: 10.1016/j.foodres.2012.08.018

[65] Aloulou A Hamden K Elloumi D Ali MB Hargafi K Jaouadi B . Hypoglycemic and antilipidemic properties of kombucha tea in alloxan-induced diabetic rats. BMC Complement Altern Med. (2012) 12:1–9. doi: 10.1186/1472-6882-12-63/TABLES/1, 22591682 PMC3403982

[66] Liu Y Zheng Y Wang W Wang Z Han S Zhou P. Kombucha enables to inhibit digestive enzymes activity and adipocyte differentiation of OP9 cells. J Food Sci. (2024) 89:10053–63. doi: 10.1111/1750-3841.17551;WGROUP:STRING:PUBLICATION, 39581587

[67] Mann FM Dickmann M Schneider R Armando S Seehusen K Hager P . Analysis of the role of acidity and tea substrate on the inhibition of α-amylase by Kombucha. J Nutr Food Res Technol. (2017):1–5. doi: 10.30881/JNFRT.00001

[68] Xu S Wang Y Wang J Geng W. Kombucha reduces hyperglycemia in type 2 diabetes of mice by regulating gut microbiota and its metabolites. Foods. (2022) 11:754. doi: 10.3390/FOODS11050754/S1 35267387 PMC8909623

